# Peripartum Maternal Admission to the Intensive Care Unit: An Observational Study over a 15-Year Period at a Tertiary Center in Austria

**DOI:** 10.3390/jcm12165386

**Published:** 2023-08-19

**Authors:** Philipp Foessleitner, Marie-Christin Budil, Stefanie Mayer, Felix Kraft, Mira Stephanie Zeilberger, Julia Deinsberger, Alex Farr

**Affiliations:** 1 Department of Obstetrics and Gynecology, Division of Obstetrics and Feto-Maternal Medicine, Medical University of Vienna, 1090 Vienna, Austria; philipp.foessleitner@meduniwien.ac.at (P.F.);; 2 Department of Anaesthesia, Intensive Care Medicine and Pain Medicine, Medical University of Vienna, 1090 Vienna, Austria; 3 Department of Dermatology, Medical University of Vienna, 1090 Vienna, Austria

**Keywords:** cesarean hysterectomy, critical ill patients, hypertensive pregnancy disorder, ICU admission, intensive care, peripartum, pregnancy, preterm birth, postpartum hemorrhage

## Abstract

Peripartum maternal admission to the intensive care unit is challenging for anesthesiologists, obstetricians, and all personnel involved. An understanding of altered maternal physiology, fetal considerations, and acute peripartum emergencies is required to ensure adequate maternal and neonatal outcomes. In this study, we analyzed data of peripartum maternal admissions to the intensive care unit at our large tertiary referral center in order to define trends and changes over time. This study retrospectively analyzed maternal morbidity, admission diagnoses, treatments, and outcomes of women with peripartum admission to the intensive care unit at our tertiary care center over a 15-year period. We found that patient characteristics and admission diagnoses remained remarkably consistent over the observational period; however, there was a significant increase in postpartum hemorrhage (r = 0.200, *p* < 0.001) and cesarean hysterectomy (r = 0.117, *p* = 0.027) over time. Moreover, we found a reduction in preterm births (r = −0.154, *p* = 0.004) and a decreased peripartum neonatal intensive care unit admission rate (r = −0.153, *p* = 0.006) among women who were transferred to the intensive care unit. Based on our long-term observational data, there is consistent need for intensive care in obstetrics due to a small number of different etiologies. Specialized training for the predominant diagnoses involved as well as multidisciplinary care of the affected patients are both warranted.

## 1. Introduction

Due to altered maternal physiology, fetal considerations, and medical emergencies associated with pregnancy, peripartum intensive care unit (ICU) admissions present a substantial challenge to anesthesiologists and involved personnel [1]. Pregnant and peripartum women account for 0.4–16% of all ICU admissions and the median incidence of peripartum admission to the ICU is 2.7 per 1000 births [1]. In the case of peripartum ICU admission, the median maternal length of hospital stay is increased from 3 to 5 days [2,3].

Although the peripartum ICU admission rate is less than 2% for industrialized countries, it is remarkably high, with rates of up to 10%, in developing countries [4,5,6]. Additionally, the mortality rate in ICUs is significantly higher in developing countries than in industrialized countries [1]. However, patient profiles and underlying diseases for ICU admission are remarkably consistent among both regions, even though admission criteria obviously vary in different hospitals, institutions, and countries [1].

Pregnant women may become critically ill due to pregnancy- and non-pregnancy-related conditions [7]. Among the most common causes for peripartum ICU admissions are exacerbation of a pregnancy-related condition, such as hypertensive pregnancy disorders, preeclampsia, eclampsia, and HELLP-syndrome, followed by hemodynamic instability, postpartum hemorrhage (PPH), and maternal sepsis [1,7,8,9]. Non-pregnancy related conditions include exacerbations of pre-existing maternal diseases, such as hypertension, diabetes mellitus, bronchial asthma, congenital cardiac defects, rheumatic diseases, and viral infections such as influenza or coronavirus disease (COVID-19) [3,10,11]. With regard to the latter, it is known that pregnant women with COVID-19 are more likely to be admitted to the ICU and suffer from severe morbidity than non-pregnant women [7,10]. Additionally, maternal age is a risk factor for peripartum ICU admission, and women who require intensive care are often at an advanced age [3]. A higher maternal age during pregnancy is associated with an increased risk of chromosomal abnormalities and spontaneous miscarriage, but also with preterm birth, stillbirth, gestational diabetes, preeclampsia, cesarean section, as well as fetal growth restriction, fetal asphyxia, and neonatal ICU (NICU) admission [12]. Nevertheless, the number of pregnancies at an advanced maternal age is constantly increasing [12,13]. In Austria, the average maternal age at delivery of the first infant increased from 23.8 years in 1984 to 30 years in 2020 [14].

In this study, we analyzed the data of peripartum maternal admissions to the ICU at our large tertiary referral center in order to analyze trends and changes over time during an observational period of 15 years. Our findings might contribute to a better understanding of maternal morbidity, which is crucial for implementing strategies to improve maternal and neonatal outcomes in the future.

## 2. Materials and Methods

The study was conducted at the Medical University of Vienna, Department of Obstetrics and Gynecology and Department of Anesthesia, Intensive Care Medicine and Pain Medicine. Data from 1 January 2007 to 31 December 2021 were analyzed to evaluate outcomes. Our hospital serves approximately 2800 to 3000 deliveries per year, including referrals from Central and Eastern Europe, and specializes in high-risk pregnancy care. Data from inpatient ICU admissions (Department of Obstetrics and Gynecology to Department of Anaesthesia, Intensive Care Medicine and Pain Medicine) and referrals from extramural centers to the ICU were considered eligible. Women who were pregnant or up to 6 weeks postpartum were considered eligible. Women younger than 18 years of age and those admitted to the psychiatric ICU were excluded.

To retrieve the data, we used the obstetric documentation system PIA Fetal Database version 5.6.28.56 (General Electric Company, GE Viewpoint, Munich, Germany), as well as the in-house hospital information system (SAP SE Inc., Walldorf, Germany). Furthermore, ICU data were gathered from digital health records using an anesthesiologic patient data management system (IntelliSpace Critical Care and Anesthesia, version J.00.01, Philips, Amsterdam, The Netherlands). Decisions on diagnosis classifications were taken by the physicians involved. Subsequently, the data were checked for plausibility and analyzed for outcomes. Maternal data including medical history, communicable and noncommunicable diseases, previous surgery, and family history were routinely collected during birth registration at our hospital. Gestational age was calculated according to the first day of the last menstruation, with possible correction for the due date during the first trimester scan. Data from external transfers were extracted by scanning the transfer letters.

We retrieved information on the basis of their importance in peripartum management, including the following: maternal characteristics; predominant indication for ICU admission; length of stay; mechanical ventilation; catecholamine requirement; use of antibiotics, erythrocyte or thrombocyte concentrates; hemodialysis; antihypertensive medication; sepsis; analgesia; and invasive monitoring. Sepsis was defined as a confirmed or suspected infection requiring vasopressors, elevated serum lactate levels, and a Sequential Organ Assessment (SOFA) score increase of ≥2 points. Retrieved data also included risk factors that are known to increase the probability of intensive care treatment, such as pre-existing hypertension, diabetes, rheumatoid disease, renal insufficiency, hyper- or hypothyroidism, pulmonary disease, cardiac disease, thrombosis/embolism, obesity, nicotine abuse, influenza or COVID-19 [15,16,17].

Additionally, the following peripartum data were retrieved: gestational age at delivery, mode of delivery (vaginal versus instrumental delivery versus cesarean section), birth weight, neonatal length and head circumference, umbilical cord pH, Apgar score, neonatal infection, NICU transfer, live versus stillbirth, and complications during pregnancy (gestational diabetes, preeclampsia, pregnancy-induced hypertension, thromboembolism, cervical insufficiency, miscarriage, and preterm birth). Moreover, we assessed whether hysterectomy was performed, the incidence of PPH, and other peripartum complications. Preterm birth was defined as iatrogenic or spontaneous delivery at or less than 36 + 6 weeks’ gestation (36 weeks plus 6 days). Stillbirth was defined as the term or preterm delivery of an infant who died in utero and was born with an Apgar score of 0/0/0.

Descriptive statistics were calculated for all the extracted variables. For continuous variables, the mean, standard deviation, and minimum and maximum values were used. Ordinally scaled variables are presented as numbers and percentages or median and interquartile range. Binary variables are presented as numbers and percentages, respectively. Depending on the data distribution, we calculated the Pearson or Spearman-Rho correlation coefficient to assess the differences in distribution over time. Correlation coefficients r ≥ 0.10 were graded as weak, r ≥ 0.30 as moderate, and r ≥ 0.50 as strong correlation. A *p*-value < 0.05 was considered statistically significant, and a *p*-value < 0.01 was considered highly statistically significant. Statistical analyses were performed using IBM SPSS Statistics for Windows, version 27.0 (IBM Corp., Armonk, NY, USA). Graphs and figures were plotted using GraphPad Prism, version 8.4.1. (GraphPad Software, San Diego, CA, USA).

## 3. Results

### 3.1. Patient Characteristics

A total of 365 patients with peripartum ICU admissions were identified and included in the statistical analyses. The median incidence of peripartum ICU admission in our cohort was 9.9 per 1000 births. Of the 365 patients admitted to the ICU, 32 (8.8%) were admitted antepartum and 333 (91.2%) were admitted postpartum. The mean maternal age at ICU admission was 32.0 years (standard deviation [SD], ±6.7). No change in maternal age at ICU admission was observed during the 15-year observational period (correlation coefficient, r = 0.093; *p* = 0.77). In our cohort, 186 women (52.2%) were primiparous at ICU admission and 43 women (12.4%) underwent assisted reproductive treatment. The most frequent pre-existing comorbidities were hyper- or hypothyroidism (20.7%), hypertension (19.5%), obesity (16.8%), and nicotine abuse (14.5%). The maternal baseline characteristics are presented in Table 1.

### 3.2. Indication for ICU Admission and ICU Treatments

**Figure 1:**
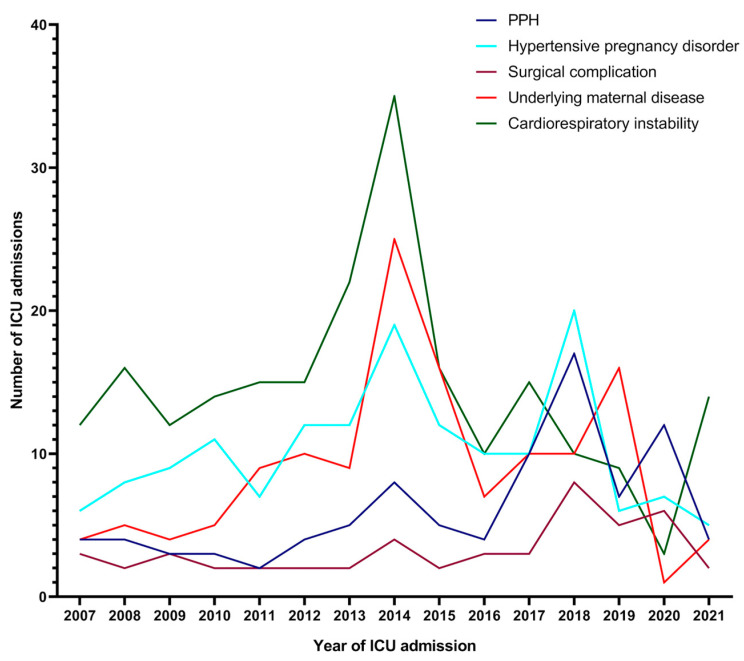
Indication for intensive care unit admission and trends over time in the 365 cases analyzed. PPH, postpartum hemorrhage.

The leading cause of ICU admission was cardiorespiratory instability in 218 patients (59.7%), followed by hypertensive pregnancy disorder (preeclampsia, eclampsia, and HELLP syndrome) in 154 women (42.2%), exacerbation of a pre-existing maternal disease in 135 women (37.0%), and PPH in 92 women (25.2%), of which 25 (27.2%) cases were caused by a placenta accreta spectrum disorder. In most cases, a combination of different indications led to ICU admission, and each indication was counted separately for analysis. We found a highly significant increase over the 15-year period with regard to PPH (r = 0.200, *p* < 0.001) and cardiorespiratory instability (r = 0.282, *p* < 0.001), which stands in contrast to the other causes that remained unchanged over time. Only one case of peripartum COVID-19 led to ICU admission at our tertiary center, due to the fact that another hospital in the region served as a center for affected patients with COVID-19 during the pandemic years of 2020 and 2021. Our patient, who was admitted in October 2020, was transferred to the ICU at gestational week 28 because of respiratory deterioration during the course of COVID-19. Extracorporeal membrane oxygenation was evaluated, but finally, the decision was made to deliver her baby by cesarean section. Postoperatively, the patient recovered quickly and was extubated on postoperative day 5. This case has also been published in the literature by Palmrich et al. [17]. Indications for ICU admission and their development over time are shown in Figure 1. The changes in the distribution are listed in Table 2.

The median duration of ICU stay was 3 days. We could not detect any significant alterations within the 15-year observational period; however, there was a mild trend towards a shorter ICU stay over time (r = −0.093; *p* = 0.08). A total of 60 women (29.1%) underwent mechanical ventilation, and 121 women (58.7%) received antihypertensive treatment. Monitoring was noninvasive in 36 women (17.6%), and invasive using blood pressure or pulmonary artery pressure in 165 women (80.5%) and 4 women (2.0%), respectively. A total of 68 women (33.2%) received erythrocyte transfusions and 38 women (18.5%) received catecholamines during their stay in the ICU. Six women (1.7%) died during their ICU stay. The intervention and treatment details are shown in Table 3.

### 3.3. Obstetric Outcomes

Pregnancy-induced hypertension was diagnosed in 107 women (30.9%), preeclampsia in 160 (45.3%), and gestational diabetes in 36 (11.6%). We did not detect any changes in the incidence of pregnancy-related complications over time in the study cohort. The mean gestational week at delivery was 32.4 (SD, ±5.4) and the mean birthweight was 1997.6 g (SD, ±1017 g). The overall preterm birth rate was 71.8%, showing a significant decreasing trend over the 15-year observational period (r = −0.154, *p* = 0.004). The live birth rate in the study cohort was 94.6%. Regarding the mode of delivery, we observed vaginal, instrumental, and cesarean section rates of 8.0%, 3.7%, and 88.4%, respectively. Therefore, we found a significant decrease in cesarean section as the mode of delivery (r = −0.138, *p* = 0.01), corresponding to a significant increase in vaginal birth (r = 0.111, *p* = 0.037) among women admitted to the ICU during the 15-year observational period. Postpartum hysterectomies were performed in 53 women (14.8%) and showed a significant increasing trend over time (r = 0.117, *p* = 0.027). Regarding the neonatal outcome, a total of 189 newborns (59.1%) were admitted to the NICU. Interestingly, we found a decrease in NICU admissions over time (r = −0.153, *p* = 0.006). Table 4 shows detailed obstetric outcomes of the study population.

## 4. Discussion

To our best knowledge, this observational study describes the data from one of the largest cohorts worldwide of women with peripartum ICU admissions. We analyzed trends and changes over time in this specific patient cohort and found a persistently high need for intensive care in obstetrics on the basis of a small number of predominant etiologies. At our tertiary center in Austria, there has been a significant decrease in preterm births, cesarean sections, and NICU admissions during the last 15 years of perinatal care.

Our incidence rate of peripartum ICU admission was 9.9 per 1000 births, which is high compared to that in previously published studies. Pollock et al. [1]. calculated an overall incidence rate of peripartum ICU admissions of 2.7 per 1000 births in a 2010 review that included 40 studies. However, the authors did not discriminate between primary, secondary, and tertiary centers; neither did they with regard to industrialized versus developing countries. Two studies analyzing data from the United Kingdom and the Netherlands reported an incidence rate of 7.6 per 1000 births, which appears to be more comparable to our results [18,19]. Interestingly, we observed a significant in-house increase in the overall incidence of peripartum ICU admission, since we had evaluated our historic data in a previous study reporting an admission rate of 6.4 per 1000 births [3]. We are aware that this increase might be attributed to a higher grade of national centralization and the establishment of our perinatal center as the “number one” high-risk obstetric clinic in Austria, with an increasing number of transfers from the entire region.

Maternal age as a risk factor for peripartum ICU admission has been controversial in the literature [1,3,20,21]. The number of women who conceive late during their lifetime is constantly increasing [12,13]. Within the 15-year observational period, we analyzed trends and changes in maternal age at ICU admission; however, we did not find a significant increase in maternal age in women with peripartum maternal ICU admission. As our study is the first of its hypothesis, our findings put this aspect into perspective.

Cardiorespiratory instability, which might be a consequence of several conditions, but also hypertensive pregnancy disorders and PPH are among the most frequent reasons for peripartum ICU admission, and their etiologies have previously been described [1,4,20,22,23]. The significant increase in cases with PPH over time that we found, as well as the corresponding increased number of cesarean hysterectomy, corresponds to an increase in PPH in the United States of 26% between 1994 and 2006 [24]. According to Corbetta-Rastelli et al., this trend was continued by a significant increase in PPH of 4.3% between 2000 and 2019 [25]. Along with this finding, the authors described a significant increase in hysterectomy due to PPH [25]. Similar results were observed in Canada between 1991 and 2004 [26]. We consider these numbers as a request for focusing on the clinical PPH management. The major cause of maternal death and severe maternal morbidity during childbirth is PPH, accounting for 27.1% of maternal demise worldwide [27]. Especially, affected regions include Northern Africa and Eastern Asia, with maternal mortality rates due to PPH of 36.9% and 35.8%, respectively. Even in industrialized countries, PPH still accounts for 16.3% of maternal demise during childbirth [27,28]. Risk factors of PPH vary in different studies and metanalyses; however, high-risk factors seem to include prior PPH, prolonged labor, multiple pregnancy, uterine rupture, cervix lacerations, placenta previa, abnormal placental invasion, artificial reproductive treatment, and fetal macrosomia [29,30,31,32].

More recent studies and meta-analyses found a significant association of hypertensive disorders with diabetes and PPH, both of which are known to be associated with vascular and perfusion abnormalities [29,30,31,33]. Considering the increasing rates of PPH [25], the main reasons for peripartum ICU admission might foster PPH risk assessment tools. The American College of Obstetricians and Gynecologists, California Maternal Quality Care Collaborative, and other institutions have introduced such risk assessment tools [34,35,36]. Therein, women are categorized into low-, medium-, or high-risk, and pretransfusion testing is being recommended [37]. These risk assessment tools have been able to identify 60–85% of patients who will later experience PPH [29,36]. Identifying these women antepartum is a key point of the risk management of PPH and its implementation into routine high-risk pregnancy care might have the potential to reduce maternal morbidity from this complication and reduce the number of cesarean hysterectomies and peripartum ICU admission at once. Although the increased number of cases of ICU admission due to PPH is alarming, it might still be a consequence of the overall small numbers in our cohort, underlying the need for a large registry study to prove or disprove this trend.

The trend of a decrease in preterm birth, cesarean section, and NICU admission that we found over time might demonstrate the success of improved peripartum management in routine high-risk pregnancy care, e.g., through routine use of an infection screen-and-treat program [38,39], use of vaginal progesterone in cases of cervical insufficiency [40,41], and the renaissance of cervical cerclage surgery [42]. A high number of measures have been introduced throughout the past decade to improve perinatal care, and we believe that our data from peripartum ICU admission cases demonstrate, at least in part, their positive effect.

The first strength of our study is the large number of cases analyzed, which are, to our best knowledge, among the largest cohorts of this specific patient population worldwide. Second, our study is the first to demonstrate how peripartum ICU care has changed over time, and we consider the 15-year observational period rather long when compared to the available literature. We were able to identify unique trends and changes over time with regard to indications for ICU admission and outcome measures.

Nevertheless, we are aware that our study has several limitations. First, it was retrospective in nature and this needs to be considered when interpreting the data. Second, since we were unable to identify an anesthesiologic evaluation score that was used over the entire study period, we could not classify changes in the anesthesiologic outcomes of the women in the ICU. We tried to evaluate the APACHE score as a tool, but data would have been available for only 4.4% of the included cases. Further studies are needed in order to analyze anesthesiologic outcomes of this specific cohort in more detail.

## 5. Conclusions

Data from our 15-year observational study in cases of peripartum ICU admission in Austria demonstrated that most patient characteristics, admission diagnoses, and obstetric outcome parameters were remarkably consistent over time. Encouragingly, we found a positive trend with regard to a decrease in preterm birth, cesarean section, and NICU admission rates among our cases. However, there seems to be a consistently high need for intensive care in obstetrics due to a small number of different etiologies. Special training for the predominant diagnoses involved as well as multidisciplinary care of the affected patients are both warranted.

## Figures and Tables

**Table 1:** Baseline maternal characteristics of 365 women with peripartum ICU admission.

Maternal Characteristic	Study Population	Maternal Characteristic	Study Population
Maternal age at ICU admission, years	32.0 (±6.7)	Duration of ICU admission, days	3 [2]
Time of admission		Assisted reproduction	
Antepartum	32 (8.8%)	Yes	43 (12.4%)
Postpartum	333 (91.2%)	No	305 (87.6%)
Parity		COVID-19 (2020–2021)	
Primipara	186 (52.2%)	Yes	1 (2.6%)
Multipara	170 (47.8%)	No	37 (97.4%)
Pre-existing hypertension		Diabetes	
Yes	69 (19.5%)	Yes	18 (5.0%)
No	285 (80.5%)	No	339 (95.0%)
Rheumatoid disease		Renal insufficiency	
Yes	16 (4.5%)	Yes	21 (5.9%)
No	340 (95.5%)	No	336 (94.1%)
Cardiac disease		Pulmonary disease	
Yes	33 (9.3%)	Yes	23 (6.4%)
No	323 (90.7%)	No	334 (93.6%)
Hyper- or hypothyroidism		Thrombosis/embolism	
Yes	74 (20.7%)	Yes	15 (4.2%)
No	284 (79.3%)	No	340 (95.8%)
Obesity		Nicotine abuse	
Yes	58 (16.8%)	Yes	50 (14.5%)
No	287 (83.2%)	No	294 (85.5%)

Data are presented as number (percentage), mean (±standard deviation), or median (interquartile range). ICU, intensive care unit; COVID-19, coronavirus disease.

**Table 2:** Trends and changes in the distribution of peripartum ICU admission over the 15-year observation period.

Indication for ICU Admission *	N (%)	Correlation	*p*-Value
Postpartum hemorrhage	92 (25.2%)	0.200	<0.001
Hypertensive pregnancy disorder	154 (42.2%)	−0.060	0.251
Surgical complication	49 (13%)	0.091	0.082
Placenta percreta/increta	31 (8.5%)	0.96	0.67
Exacerbation of maternal disease	135 (37.0%)	0.014	0.797
Cardiorespiratory instability	218 (59.7%)	0.282	<0.001
Thrombosis/PE	19 (5.2%)	0.058	0.266
Infectious disease (excl. COVID)	22 (6.0%)	0.008	0.872

* In most cases, a combination of causes led to the ICU admission; therefore, the percentage exceeds 100%. N = number, % = percentage. The correlation depicts the difference of distribution over the 15-year observation period. ICU, intensive care unit; COVID-19, coronavirus disease; PE, pulmonary embolism.

**Table 3:** Treatments and interventions of 365 women with peripartum ICU admission.

Treatment/Intervention	N (%)	Treatment/Intervention	N (%)
Mechanical ventilation	60 (29.1%)	Thrombocyte concentrate	17 (8.3%)
Catecholamines	38 (18.5%)	Hemodialysis	10 (4.9%)
Antibiotics	103 (50.0%)	Antihypertensive treatment	121 (58.7%)
Erythrocyte concentrate	68 (33.2%)	Analgesia	193 (94.1%)
Anesthesia		Monitoring	
General	20 (9.7%)	Non-invasive	36 (17.6%)
Spinal	3 (1.5%)	Invasive	165 (80.5%)
None	183 (88.8%)	Pulmonal artery	4 (2.0%)

N = number, % = percentage. ICU, intensive care unit.

**Table 4:** Obstetric outcomes of 365 women with peripartum ICU admission.

Obstetric Outcome	Study Population	Obstetric Outcome	Study Population
Gestational week at delivery	32.4 (±5.4)	Length (cm)	43.4 (±7.8)
Birthweight (grams)	1997.6 (±1017)	Umbilical cord pH	7.25 (±0.1)
Preterm birth Yes No	250 (71.8%) 98 (28.2%)	Cervical insufficiency Yes No	36 (10.3%) 312 (89.7%)
Pregnancy-induced hypertension Yes No	107 (30.9%) 239 (69.1%)	Preeclampsia Yes No	160 (45.3%) 193 (54.7%)
Gestational diabetes Yes No	36 (11.6%) 314 (89.7%)	Postpartum hysterectomy Yes No	53 (14.8%) 305 (85.2%)
NICU admission Yes No	189 (59.1%) 131 (40.9%)	Neonatal demise Yes No	28 (8.4%) 307 (91.6%)
Delivery mode Vaginal Instrumental Cesarean section	28 (8.0%) 13 (3.7%) 311 (88.4%)	Apgar score at 5 min 0–4 5–8 9–10	25 (7.5%) 86 (25.8%) 222 (66.7%)

Data are presented as number (percentage) or mean (±standard deviation). ICU, intensive care unit; NICU, neonatal intensive care unit.

## Data Availability

Full data are available upon reasonable request to the corresponding author.
